# A Review of Diet and Foraged Pollen Interactions with the Honeybee Gut Microbiome

**DOI:** 10.1007/s00248-025-02551-y

**Published:** 2025-05-27

**Authors:** Dara Eoin Meehan, Paul W. O’Toole

**Affiliations:** School of Microbiology & APC Microbiome Ireland, University College Cork, Cork, Ireland

**Keywords:** Honeybee, *Apis mellifera*, Microbiome, Pollinator, Metabolism, Diet

## Abstract

The honeybee *Apis mellifera* is a globally vital pollinator for flowering plants and crops, but it is currently facing mounting threats to survival due to habitat anthropization, emerging pathogens, and climate change. Over the past decade, increasing research efforts to understand and combat these challenges have led to an exploration of the honeybee gut microbiome—a relatively simple and highly conserved community of commensals which has a range of effects on the host. Researchers have now unravelled the main functional roles of this microbiome which include innate immune system stimulation, metabolism of dietary compounds, and mediation of host development and behaviour. Key amongst these is its role in aiding nutrition through the metabolism of complex carbohydrates and by degradation of otherwise indigestible pollen compounds. Increasingly, research is indicating that a diverse and high-quality pollen diet is key to maintaining healthy colonies and a stable microbiome. However, colonies can struggle to meet these dietary needs, particularly if they are located in anthropized ecosystems. Disruptions to honeybee diets or a reduction in the availability of diverse foraging options can significantly alter the composition of the microbiome, shifting it towards an abnormal state that leaves the honeybee more vulnerable to infection. Seasonal changes, primarily the overwintering period, also induce shifts in microbiome composition and are periods of time when a colony is particularly vulnerable to pathogenic infection. A comprehensive understanding of the effect these variables have on both microbiome composition and colony health is key to tackling the unprecedented environmental challenges that honeybees now face. This review summarises recent research which has elucidated the functional role of the gut microbiome in metabolism and how the composition of this bacterial community can alter due to seasonal change, anthropized landscapes, and dietary shifts. Finally, we also discuss recent studies investigating the effect that dietary supplementation has on the gut microbiome and the application of probiotic candidates for improving colony resilience and strength.

## Introduction

Over the last 15 years, significant efforts have been made to elucidate the taxonomic composition and functional roles of the honeybee gut microbiome, with a specific focus on the worker microbiome [1–12]. This microbiome is significantly different from that of other honeybee castes [13, 14] and is both relatively simple [11] and highly conserved [7]. It is acquired primarily after new workers emerge in the hive environment, through contact with nurse bees and their surroundings [15, 16]. This review summarises the current understanding of the role of the microbiome in metabolism and critically analyses studies investigating how forage (diet) and ecosystem interact with the honeybee microbiome. Additionally, we highlight recent studies investigating the effects of dietary supplementation and the application of probiotic taxa to alleviate dysbiosis of the microbiome and improve honeybee health.

The honeybee gut microbiome is usually defined by the presence of a small group of bacterial phylotypes, broadly considered to be the ‘core’ microbiome, and predominantly located in the hindgut [1, 3, 17]. While there is no formal definition of which taxa are considered core and non-core, it is broadly agreed that the ‘core’ microbiome is comprised of 5 genera which are present in all worker bees: *Bifidobacterium*, *Bombilactobacillus*, *Lactobacillus*, *Gilliamella*, and *Snodgrassella* [1]*.* Other genera which are often found in the gut, but which are not considered members of the ‘core’ microbiome, include *Bartonella*, *Commensalibacter*, and *Frischella* [1]. These non-core taxa are usually present in lower abundance compared to core taxa, but can proliferate as a result of seasonal change [18], dietary changes [19], pathogenic infection [20], or features in surrounding ecosystems [21].

These core and non-core taxa have a broadly symbiotic relationship with the honey bee host: contributing to the metabolism and fermentation of carbohydrates [8, 22, 23], innate immune system stimulation by upregulating the production of antimicrobial peptides [24, 25], and development and weight gain by triggering changes in vitellogenin expression and insulin signalling [26]. They also protect the honeybee host directly against pathogenic microorganisms either through competitive inhibition [27] or the production of antifungal metabolites [28]. Recently, they have even been linked to the breakdown of pesticides by upregulating host expression of cytochrome P450 enzymes, a key component of detoxification pathways [29].

Perturbations of the microbiome can alter the composition and relative abundance of taxa (sometimes called *dysbiosis*) which can in turn negatively affect host health and fitness [3, 12, 19]. States of dysbiosis are primarily caused by pathogenic activity [20, 30], pesticide exposure [20, 31], and antibiotic treatment [32]. Typically, a dysbiotic honeybee microbiome is defined by alterations in alpha-diversity and changes in the relative abundance of core and non-core taxa [19, 32]*.* Extensive reviews have documented how different factors alter the gut microbiome and affect the health of the honeybee [1, 3, 12, 33, 34]. As understanding of this complex relationship between honeybee host and commensals deepens, studies are currently exploring how additional factors such as diet [19, 35], seasonal change [18, 36], and surrounding ecosystem [36–38] interact with the microbiome. An improved understanding of how both ecosystem and diet can shape the microbiome is vital to alleviate the stresses of agricultural intensification and climate change.

## The Functional Roles of Gut Commensals in Metabolism

**Fig. 1:**
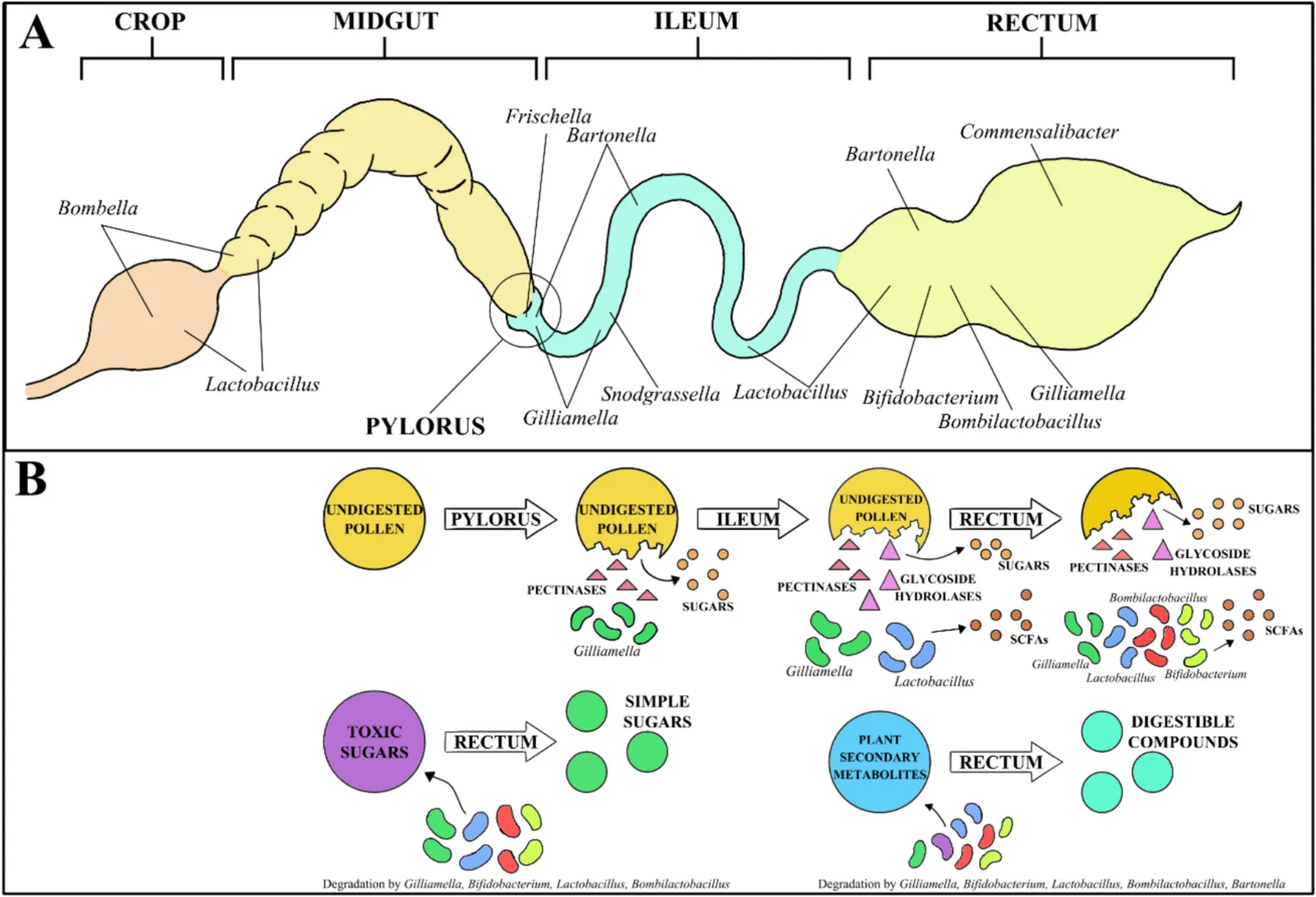
Locations and metabolic functions of the honeybee gut microbiome. **A** The honeybee digestive tract, with the typical locations of core and non-core taxa of the gut microbiome indicated. **B** The role of core gut taxa in metabolism of indigestible carbohydrates, toxic sugars, and plant secondary metabolites

The diet of adult honeybee workers is relatively simple, consisting primarily of honey and beebread which serve as the main sources of carbohydrates and protein [39, 40]. Recent studies have shown that a diverse and high-quality pollen diet can enhance both bee physiology and immunity [41, 42] and that time-of-year affects the quantity and diversity of foraged pollen [43]. Research is now exploring the complex role that the gut microbiome plays in the metabolism of pollen, and the influence of diet on microbiome composition [19, 23, 35, 44, 45]. As summarised graphically in Fig. 1, these data indicate that the members of the core microbiome primarily contribute to the metabolism of indigestible carbohydrates and the fermentation of resulting products [8, 10, 22, 46–48]. This metabolic activity has a positive effect on honeybee physiology, primarily by promoting whole body growth and weight gain by contributing to host nutrition [26]. Here, we summarise the current understanding of how gut microbiota contribute to host digestion through the metabolism of indigestible compounds, niche specialisation within this community and its role in detoxifying potentially harmful sugars.

### Core Taxa of the Microbiome Metabolise Complex Carbohydrates and Pollen Derivatives

The predominant expression of genes for carbohydrate metabolism and transport systems within several of the core taxa [8, 17, 48, 49], suggests that the honeybee gut is colonised by bacteria which are well-adapted to their environment. The processing of pollen into beebread and its digestion by worker bees involves the breakdown of complex carbohydrates by glycoside hydrolases, secreted by the hypopharyngeal glands in the worker head [45]. The resulting simple sugars such as glucose and fructose are absorbed in the midgut [50], with undegraded dietary compounds, namely, components of the pollen cell wall, passing to the hindgut where they are metabolised by gut microbiota [46, 48].

Metagenomic, metatranscriptomic, and experimental colonisation studies show that members of the core microbiome (*Lactobacilli*, *Bombilactobacilli*, *Bifidobacteria*, and *Gilliamella*) have significant fermentative capabilities within the honeybee gut, producing short-chain fatty acids (SCFA) lactate, succinate, formate, and acetate [8, 46, 48, 49]. The core taxa metabolise complex carbohydrates and components of the pollen wall by secreting glycoside hydrolases, pectinases and polysaccharide lyases [47, 51]. Saccharolytic fermentation is understood to be the predominant metabolic activity of the gut microbiota [26, 48] and its products (SCFAs) are utilised by both the host and other microbiota and associated with hindgut physiochemical conditions [26].

### Specialisation in Metabolic Activity Within the Gut Microbiome Community

While there is overlap in the metabolic function of members of the gut microbiota [48], certain taxa have unique roles and niche specialisation, depending on their enzymatic capabilities and location within the hindgut [8, 47, 48, 51]. For example, specific *Gilliamella apicola* strains degrade pectin from pollen cell walls by the production of pectinases [47], whereas certain honeybee-associated *Bifidobacterium* strains display an enriched repertoire of glycoside hydrolase genes, including GH43, enabling them to metabolise hemicellulose [47]. Furthermore, *Lactobacillus* strains within a species show extensive inter-strain variation in genes encoding phosphotransferase systems and glucose hydrolases [51], displaying niche specialisation within the honeybee gut by metabolising different pollen-derived substrates [46].

### Degradation of Toxic Compounds and Metabolism of Plant Secondary Metabolites

The gut microbiota can metabolise sugars which are toxic to the honeybee host such as xylose, arabinose and mannose [23, 45, 51, 52]. In particular, *Gilliamella apicola* strains possess a variety of genes for metabolising toxic dietary carbohydrates [23], and *Lactobacillus* and *Bifidobacterium* contain clusters of gene associated with the transport and fermentation of mannose [51, 53]. A recent study also describes how *Bifidobacterium*, *Bombilactobacillus*, and *Giliamella* strains degrade amygdalin, a secondary metabolite from almond pollen which, without degradation by gut microbiota, accumulates as prunasin in the midgut and hindgut [52]. Similarly, non-core taxa *Bartonella apis* and *Apilactobacillus kunkeei* have recently been shown to improve resistance to toxic nectar sources [54]. While the underlying mechanism is unclear, it is hypothesised that these non-core taxa improve resistance through the upregulation of pleiotropic honeybee-associated immune genes.

Other recent studies have identified members of the gut microbiome that are capable of utilising and degrading plant secondary metabolites found in nectar and pollen [46, 48, 55]. In particular, *Lactobacillus* and *Bombilactobacillus* can metabolise plant secondary metabolites such as flavonoids, located in the outer pollen coat [46, 48], contributing to the breakdown of undigested pollen. Non-core taxa such as *Bartonella* are also capable of degrading plant secondary metabolites [54] and have a wider range of energy pathways [56] which could explain their ability to thrive in stressful conditions such as overwintering [56] or drought [57]. However, the beneficial effects of the degradation of plant secondary metabolites by members of the gut microbiome are not yet fully clear and require further investigation.

Overall, these studies highlight one of the key functional roles of the honeybee gut microbiome: the metabolism of dietary compounds which otherwise could not be utilised by the honeybee host or microbiota (Fig. 1). Through the production of SCFAs, monosaccharides, and degradation of potentially toxic sugars, the microbiome contributes to overall host health and physiology. While the community of microorganisms within the gut is relatively small, niche specialisation allows these taxa to co-exist. This community is, however, vulnerable to compositional shifts induced by changes in the honeybee diet. In the following section, we will discuss how suboptimal and nutritionally deficient honeybee diets can have a significant effect on the composition of the microbiome and lead to dysbiosis.

## Variation in Pollen Diet and How It Interacts with the Microbiome

The importance of nutrition in honeybee health has been extensively studied and recently reviewed [58], emphasising that a diverse pollen diet supports overall health and pathogen resistance [40, 59, 60]. Given the role of the gut microbiome in metabolism, it is logical to hypothesise that there could be an interaction between changes in diet and the composition and functional roles of the microbiome. Recent studies have begun to examine this interaction in honeybees [19, 61–65] and other social and solitary bees [66]. Collectively, they indicate that variation in the quality and type of pollen can influence microbiome composition, bee health, and pathogen resistance.

### Pollen Abundance and Quality Affect Microbiome Composition

In general, honeybees appear to have a preference for fresh pollen over older, stored pollen [117], and it has been shown that the age and quality of pollen also has a significant role in shaping the gut microbiome of developing workers [19]. In this study [19], young bees consuming a diet of aged pollen developed a dysbiotic microbiome characterised by the proliferation of opportunistic non-core microbiota *Frischella perrara* and *Bombella apis* (formerly *Parasaccharibacter apium*) and a reduction in proportional abundance of *Snodgrassella alvi*. This diet also correlated with higher *Nosema* spp. spore counts, indicating a vulnerability to pathogenic infection. Interestingly, in this study, bees fed a commercial pollen substitute (of undefined composition) showed no significant alterations in microbiome composition compared to those fed fresh or aged pollen. This contrasts with a recent study [62] which tested the effect of a diet designed to mimic the macronutrient profile of *Brassica rapa* pollen mixed with sucrose, cellulose, and vitamins on the microbiome. This study concluded that the pollen-substitute diet reduced microbiome alpha-diversity and impaired the host’s ability to supress opportunistic pathogens. However, they also note that the core taxa of the microbiome remained relatively stable when bees fed upon pollen-substitute diets and the shift in alpha-diversity was primarily due to alterations in the abundance of non-core taxa.

Pollen starvation also disrupts microbiome composition and metabolic function [65], whereby pollen-deprived bees exhibit significant reductions in the abundance of core taxa, *Lactobacillus*, *Bombilactobacillus*, *Bifidobacterium*, *Gilliamella*, and *Snodgrassella.* This is accompanied by a reduction in the expression of fermentative enzymes, highlighting a compromised metabolic capacity.

### A Diverse Pollen Diet Is Vital for Honeybee Health and Microbiome Stability

Pollen diversity (polyfloral/monofloral diet) has a significant effect on the composition of the gut microbiome [64]. In a comparison of polyfloral pollen diet *versus* monofloral (*Eucalyptus grandis*) pollen, a monofloral diet resulted in lower proportional abundance of *Lactobacillus*, *Bombilactobacillus*, and *Bifidobacterium* alongside an increase in the abundance of *Bartonella apis*. Consistent with previous studies [19], the monofloral diet resulted in increased prevalence of *Nosema* spores. Monofloral pollen diets have previously been shown to have detrimental effects on overall honeybee health, specifically in their ability to combat pathogenic infection [42, 67]. This highlights the complex relationship between diet, health, and microbiome composition in honeybees—a lack of diversity in foraged pollen appears to drive dysbiosis [64] and given the role of the microbiome in honeybee immunity [25] and reduces the host ability to supress infection.

These studies collectively explain how variation in pollen diet can disrupt the composition of the honeybee gut microbiome, leading to declines in the abundance of core microbial taxa and the proliferation of opportunistic colonisers. Diets of aged or monofloral pollen and pollen starvation all have detrimental effects on the composition of the gut microbiome and, thus, negative consequences for host health. As outlined by Dolezal and Toth [58], the interplay between diet, disease, and the microbiome is complex; disruptions in one of these variables can initiate a feedback loop with cascading negative effects on honeybee heath. Their review clearly outlines the nutritional stresses that honeybees face as a result of human-mediated landscape changes. However, relatively few studies have focused on the specific interaction between pollen diets and microbiome in honeybees. Considering the growing pressures from agricultural intensification and widespread monoculture practices [68], a better understanding of this interaction is needed to mitigate the challenges facing honeybee colonies. In the following section, we will review the effect of seasonal change and anthropized ecosystems on honeybee health and microbiome composition.

## Ecosystem and Seasonal Change Affect Forage Availability and Microbiome Composition

The composition of the local ecosystem and seasonal change influence the availability of pollen and nectar to honeybees which impacts their diet and thus the composition of their microbiome. As colonies transition from summer to winter, their microbiome also changes [18]. Usually, these changes are characterised by a shift in the abundance of core taxa and the proliferation of certain non-core taxa such as *Bartonella* [56]. It is likely that this shift in microbiome composition is a response to the winter dietary change, and it also marks a period of time where colonies are more vulnerable to pathogenic infection [69]. Additionally, recent studies are now highlighting the detrimental effect that anthropized landscapes have on the availability of nutritional resources [68] and the composition of the honeybee gut microbiome [21]. While this is a relatively new area of research, trends are emerging which suggest that honeybee colonies located in areas of intensive monoculture or urbanised ecosystems are more prone to disease [41] and have an atypical microbiome composition [37]. In this section, we discuss how both seasonal change and local ecosystem can affect the composition of the gut microbiome and colony health.

### The Composition of the Gut Microbiome Changes with Season

Between seasonal change and local ecosystem, the effect of the former on the microbiome is better understood, with many studies identifying a change in the relative abundance of core taxa [18, 36, 56, 70] and total bacterial abundance [18] as colonies progress through the honey season and enter the overwintering period. The overwintering shift is primarily characterised by a decrease in alpha diversity [18] and increased abundance of certain core taxa such as *Gilliamella* and *Snodgrassella* [18, 71] and non-core taxa such as *Bartonella* and *Frischella* [18, 56, 69]. These changes are likely driven by a combination of altered diet, temperature change, and the emergence of longer-living overwintering bees in the hive. In particular, the proliferation of *Bartonella* during this period may be associated with dietary changes, as its wider range of energy pathways allows it to circumvent the reduced availability of pollen and utilise metabolic waste products for energy [56].

It is a common practice for beekeepers to provide supplemental feed for their colonies during the overwintering period, usually in the form of commercial sugar fondant or syrup solutions, and this can affect the composition of their microbiome [72, 73]. In this case, core taxa typically remain stable but their relative abundance is affected by feed type. Notably, there is an increase in the relative abundance of *Bartonella* and *Bifidobacteria* when fed with specific syrup solutions [71–73]. An increase in abundance of these taxa is not indicative of dysbiosis and actually appears to increase the likelihood of a colony surviving the overwintering period [69, 72].

The composition of the overwintering microbiome is also a predictor of colony survival during this period [69]. Core-taxa *Gilliamella* and *Snodgrassella* are positively associated with survival, and colonies which survive the overwintering period typically have a higher total abundance of bacteria in their gut. Interestingly, non-core taxa including *Commensalibacter*, *Bartonella*, *Bombella*, and *Frischella*, sometimes considered opportunistic colonisers, are also present in greater abundance in colonies which survive the winter. The proliferation of these non-core taxa could be linked to dietary changes, namely, the consumption of older, stored beebread, as has previously been noted for bees fed a diet of aged pollen [19]. Interestingly, *Snodgrasella* abundance in the overwintering microbiome shows inconsistent results across studies, with some reporting an increase in its abundance [69, 71] while others describe a decrease in abundance [18, 73]. *Commensalibacter* has previously been associated with thriving colonies [2] and an increase in its abundance has been observed in the overwintering microbiome in other studies [18], though it is not usually considered a member of the core microbiome. Thus, there are some contradictory findings on this topic, probably due to unrecognised local factors or confounders.

Seasonal changes may also allow opportunistic pathogens to proliferate in the microbiome, with the transition to the overwintering period being a particularly vulnerable period for colonies. For example, two separate studies [36, 71] observed an increase in *Arsenophonus* abundance during the early wintering period. While the exact effect of this genus on honeybee health is unclear, studies have shown that its presence in the honeybee microbiome is associated with *V. destructor* infestation [118] and an increased abundance of the genus has been linked to dysbiosis [7] and Colony Collapse Disorder [74].

The overwintering period brings about significant change for honeybee colonies with the emergence of longer-living bees, a dietary shift to stored pollen and/or supplemental feed, and a period of dormancy when bees rarely leave the hive. It now appears that seasonal change also has a significant effect on the composition of the microbiome. While the core taxa are retained, their abundance can change as non-core taxa such as *Frischella*, *Commensalibacter*, and *Bartonella* proliferate [18, 56, 69]. This microbiome, which is significantly different on a compositional level from the summer gut microbiome [18], likely has a role in honeybee health and survival during this time period [69]. It should be noted that, in addition to changes in overwintering bee physiology, these alterations in bacterial abundance could be driven by a range of factors including dietary supplementation, temperature change, emergence of long-living bees, or their state of dormancy. Future studies should focus on elucidating exactly which variables are driving the proliferation of specific species, perhaps facilitated by studying (non-supplemented) wild honeybee hives.

### Anthropized Ecosystems Significantly Influence Forage Availability and Diet and Are Detrimental to Honeybee Health

**Fig. 2:**
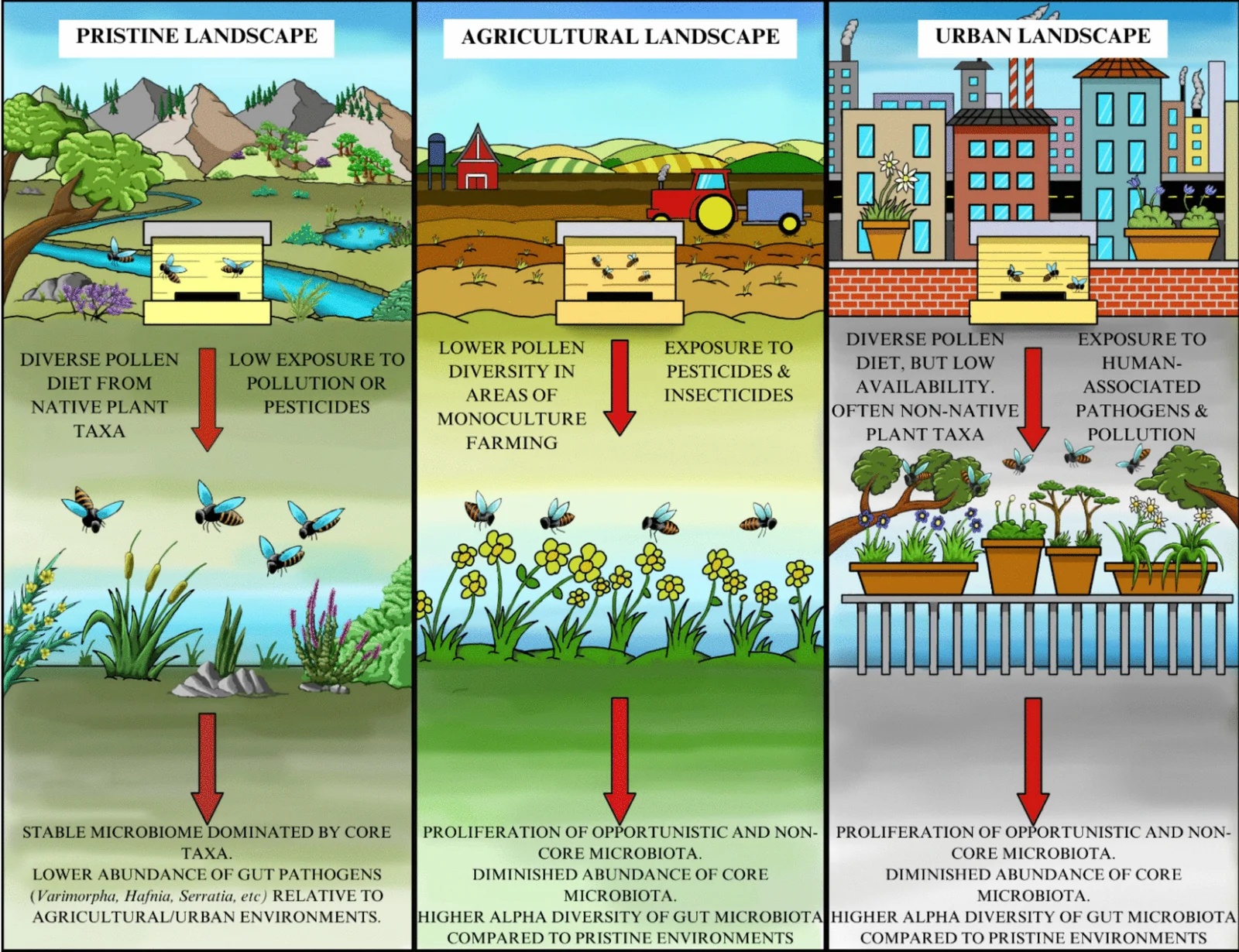
The effects of different landscape types on honeybee health and gut microbiome stability

Understanding the effect that the surrounding ecosystem has on the honeybee microbiome is a more challenging task. Anthropized changes to the landscape have an impact on the health of honeybee colonies, particularly agricultural areas and urbanised centres (as summarised graphically in Fig. 2). Agricultural landscapes affect the availability and diversity of pollen and nectar for honeybees [75, 76], impacting the quality of their diet [41, 77] and the nutritional composition of beebread [68]. Additionally, agricultural landscapes can expose honeybee colonies to a variety of pesticides such as imidacloprid, thiamethoxam, clothianidin, and acetamiprid, which have significant impacts on honeybee microbiome, as recently reviewed [31, 33, 78]. Forager flight range is also affected by the surrounding landscape, primarily determined by the occurrence and diversity of flowering plants in their vicinity [43, 79, 80].

In contrast, honeybee colonies located in urban or suburban settings have more diverse foraging options compared to those in agricultural landscapes [81], often foraging from a variety of plant species including non-native taxa [82, 83]. This is likely due to lower overall plant availability, resulting in bees foraging from urban gardens/parks and making foraging a more challenging and energy intensive task (Fig. 2). Notably, a recent study [84] found that, when located adjacent to both urban and agricultural landscapes, foragers will preferentially collect pollen from the agricultural area.

A recent investigation [85] which compared colony strength and levels of *Nosema* infection between colonies located in urban and agricultural landscapes concluded that colonies located in urban environments were stronger and had lower levels of *Nosema* infection compared to those in agricultural environments. A diverse pollen diet is reportedly linked to healthier colonies [67], a stable microbiome and lower *Nosema* infection [64]. However, these results are contradicted by the findings of another investigation [37] which found that colonies located in urbanised environments harboured more pathogenic bacteria and fungi compared to those in natural or agricultural environments. These conflicting outcomes could be explained by variation in the floral composition and pollen availability between these urbanised settings.

### Honeybee Gut Microbiome Composition May Be Detrimentally Affected by Anthropized Landscapes

Currently, few studies have examined the direct effect of the surrounding landscape on the microbiome, making it challenging to draw conclusions on how colony location impacts microbiome structure. In general, the honeybee microbiome is understood to be highly conserved across geographical locations [6, 11, 86] and some microbiome studies which have examined this have found only marginal differences [87] or no variation [36] in microbiome composition across geography. However, recent studies which have investigated anthropized/non-anthropized landscapes and their effect on the honeybee microbiome have found that the relative abundance of core and non-core taxa can differ depending on their surrounding ecosystem [21, 37, 38, 88, 89].

At present, research in this area has predominantly focused on the effect of agricultural landscapes on the composition of the microbiome [21, 38, 88, 89]. These studies have primarily concluded that anthropized agricultural areas have a detrimental effect on the microbiome and are associated with the proliferation of opportunistic taxa and diminished abundance of core taxa. The microbiomes of colonies located in agricultural landscapes tend to have higher alpha-diversity compared to those located in pristine or natural environments [21, 89], primarily due to a greater abundance of these non-core taxa. Notable amongst these are *Bartonella* [21, 38] and *Enterobacteraceae* [89] which are more abundant in these agricultural landscapes, while core taxa such as *Lactobacillus*, *Snodgrassella*, and *Gilliamella* are enriched in pristine/natural habitats [21, 89]. While the majority of these studies did not measure colony strength or the levels of known pathogens, the findings correlate with previously mentioned studies into the effect of monofloral diets on the honeybee microbiome [64] and suggest that colonies in these agricultural landscapes could be more vulnerable to pathogenic infection.

There has been little research conducted into the effect of urbanised landscapes on the honeybee microbiome. One study which investigated the effect of oxalic acid (a common treatment for *Varroa destructor*) on *Apis m. iberiensis* colonies located in agricultural, mountainous, and urbanised landscapes found that, before any treatment was applied, microbiome composition was significantly different in the urbanised landscape compared to agricultural or mountainous [37]. This difference was primarily attributed to the enrichment of non-core and pathogenic taxa (*Bartonella*, *Hafnia alvei*, and *Serratia*) in urbanised colonies, resulting in overall higher alpha-diversity. Additionally, higher levels of *Nosema* were detected in these colonies which, along with the presence of other pathogenic taxa (*Hafnia alvei* and *Serratia*), suggests a state of dysbiosis that was not observed in rural or mountainous locations. Interestingly, this contradicts previously mentioned investigations [85] which found that *Nosema* spores were less abundant in urbanised landscapes compared to agricultural, though differences in the exact composition of the urbanised landscape in both studies could be a likely contributor to the differences in results. Notably, the least anthropized landscape in this study (mountainous) had the lowest abundance of pathogens, possibly attributable to a combination of diverse natural forage and stable microbiome.

Despite the relatively small number of studies which have investigated the effect of landscape on the microbiome, trends are emerging which suggest that in anthropized landscapes, the honeybee microbiome is perturbed, with higher abundance of non-core taxa and diminished abundance of core taxa (Fig. 2). The exact impact of this compositional shift on the overall health of the honeybee is not yet fully clear. Future studies should focus on a combined analysis of microbiome composition and colony strength/health, examining how they vary between agricultural, natural, and urbanised landscapes.

## Dietary Supplementation for Honeybees

Honeybee colonies are often provided with dietary supplements by beekeepers at the beginning of the overwintering period or during periods of pollen shortage. In areas where honeybee colonies are used to pollinate early-season crops, supplementation is also provided during winter in order to strengthen colonies before deployment to pollination service [63, 90]. Dietary supplements are typically intended to provide a source of either carbohydrates or protein to a colony in the absence of nectar or pollen. Many studies have investigated their effects on colony strength [63, 91, 92] and health [93], with recent reviews highlighting key findings [40, 58, 90]. These provide a particularly comprehensive overview of the benefits and disadvantages of dietary supplements, highlighting that while there is some discrepancy between different studies, supplements generally increase brood and worker population but are less effective when environmental pollen and nectar are plentiful. This underscores the fact that, while dietary supplementation can benefit a colony during challenging environmental conditions, natural pollen is superior to supplementation. There is inherent complexity involved with carrying out studies on the effect of dietary supplementation on colonies, namely, discrepancies between caged and field trials, differences in supplement composition, the effect of landscape/available forage and low sample sizes. The following section of this review presents an overview on emerging research concerning the effect of such dietary supplements on the honeybee microbiome.

### Dietary Supplements Can Cause Slight Changes in the Composition of the Gut Microbiome Community

Until recently, there has been a lack of research which has examined the effect of dietary supplements on the honeybee microbiome. However, in the last 5 years, there has been an increased number of studies which have investigated possible interactions between these supplements and gut microbiome composition [55, 62, 63, 72, 94–98]. These studies primarily conclude that the application of dietary supplements does not alter the presence of core commensals in the gut, though their abundance can be affected [55, 72, 95, 98]. Additionally, non-core taxa which are normally present in low abundance can proliferate as a result of dietary supplementation [96, 98].

Shifts in the abundance of gut taxa after dietary supplementation may be subtle [63, 96] and depend on the type of supplementation applied. Thus far, studies have mainly focused on the effects of commercial supplements designed to mimic the nutritional profile of a natural diet [63, 96], and carbohydrate supplements which are usually applied in a syrup solution [72, 98] or pollen substitutes [62]. Sucrose supplementation, commonly applied to colonies during overwintering or dearth periods, results in an increase in the abundance of *Bifidobacteria*, *Bartonellaceae*, and *Lactobacillus* [72, 73, 98]. Given the fermentative capabilities of these taxa [48], it is logical that they would proliferate with a diet rich in simple sugars. Supplements designed to mimic the macronutrient profile of pollen appear to have a negative impact on the composition of the microbiome, reducing its diversity and evenness and reducing its ability to resist pathogenic infection [62]. Commercial supplements differ greatly in their nutritional content [63] and so it is impossible to broadly define their impact on the gut microbiome. However, the few studies which have examined these commercial alternatives and their effect on the microbiome have so far found that they have little impact on its composition [63, 96]. Notably different to these, one study investigated the effects of phytochemical supplementation on the microbiome, reporting that it had a positive effect on composition, increasing the abundance of *Snodgrassella*, *Commensalibacter*, and *Lactobacillus* spp. [55].

Difference in experimental design between these studies is an important consideration when interpreting these results. As mentioned above and highlighted in previous reviews [90], confounding variables such as surrounding landscape can obscure the effect that dietary supplementation has on colony performance and microbiome composition. These effects must be considered in field studies, because experimental colonies will be exposed to variables such as pathogenic infection, differing management practices and landscape/seasonal influence. Notably, recent research concluded that landscape has more of an impact on gut microbiome composition than does supplementation [63], highlighting the relative effect that different variables have on the gut microbiome. Seasonal influence and changes in climate must also be considered, particularly as the composition of the gut microbiome varies between winter and summer [18]. Notably, dietary supplementation appears to have little effect on the gut microbiome when applied during the foraging period [96] but a significant impact when applied during the overwintering period [72].

Similarly, comparisons between studies can be difficult to make if they differ in design between cage trials [55, 62, 94, 98] and field studies [63, 72, 96]. Laboratory studies remove the influence of additional variables and allow for the examination of a single factor such as dietary supplementation, but recent work has shown that these caged experiments themselves effect honeybee immune function and microbiome composition [99, 100] and are not representative of in-hive environments. Ideally, studies should combine caged and field trials when examining the effect of dietary supplements [91] but this is not always possible. Therefore, a cautionary approach should be taken when drawing comparisons between multiple studies that vary between cage and field trials.

### Probiotics for Honeybee Colonies—Differences Between Native and Non-native Candidates

Finally, dietary supplementation with ‘probiotic’ taxa is emerging as a particularly interesting area of research and has been comprehensively reviewed in several recent publications [101–104]. Many probiotics currently available to beekeepers are a combination of bacterial taxa which are not native to the honeybee gut [101]. Reports on the efficacy of these probiotics do not always concur [105, 106], with recent studies highlighting minimal or no beneficial effects for colonies treated with non-native probiotics [107, 108]. In some cases, they even reduce colony ability to combat infection and increase mortality [109]. Conversely, other studies have reported positive outcomes when colonies have been treated with commercial or non-native probiotics, such as a reduction in *Nosema* spore numbers [119, 120], increased capacity to combat American Foulbrood [105], increased honeybee lifespan [121] and ability to recover from antibiotic-induced dysbiosis [122]. Additionally, recent studies have begun to explore the concept of applying native gut taxa as probiotic agents, suggesting that formulations comprised of these taxa can have beneficial effects [110–113]. This emergent area of research shows potential, though more work is needed to evaluate the effect of these probiotic candidates in field trials, which do not always mirror laboratory results [114]. In particular, while laboratory cage trials often demonstrate reduced pathogen loads following the application of probiotic treatments, low-level infection can still persist, which could partially explain discrepancies in results between cage and field trials. The method for delivery of probiotic treatments to colonies is also an important consideration which must be factored into any field trial which seeks to evaluate the efficacy of native taxa as probiotic candidates [115, 116].

## Conclusion

This review highlights the complex interactions between honeybee diet, local ecosystems, microbiome composition, and colony health. Over the past decade, substantial advances have been made in understanding the composition of the honeybee gut microbiome and its symbiotic relationship with the host. A critical function of this highly conserved microbial community is the metabolism of dietary compounds indigestible by the honeybee, including complex carbohydrates and pollen cell wall components. The niche specialisation of gut commensals has resulted in a stable and highly adapted microbial consortium, despite the relatively limited diet of the honeybee.

Recent research has turned towards establishing methods to alleviate the pressures of anthropized ecosystems, where agricultural and urban landscapes negatively impact honeybee health and microbiome composition. While evidence suggests these detrimental effects are primarily driven by the availability and diversity of forage, the specific factors which lead to microbial dysbiosis remain poorly understood. Nutritional stress likely plays a pivotal role, and disruptions in diet can create feedback loops that increase vulnerability to pathogens and other stressors.

Dietary supplementations, including pollen substitutes and probiotics, offer a promising alternative to conventional feeds and antibiotics, to alleviate environmental and pathogenic pressures. However, current research in this area remains inconclusive as to the efficacy of such treatments. Continued efforts are needed to refine supplementation strategies that stabilise or enhance microbiome function and improve colony performance/health under environmental stress. Additionally, studies that identify pollen sources associated with healthy colonies could inform sustainable development initiatives and guide the planting of pollinator-friendly vegetation. These approaches collectively hold the potential to mitigate the challenges posed by agricultural intensification and urbanisation, supporting the long-term resilience of honeybee populations.

## Data Availability

No datasets were generated or analysed during the current study.
